# The role of oviduct-specific glycoprotein (OVGP1) in modulating biological functions of gametes and embryos

**DOI:** 10.1007/s00418-021-02065-x

**Published:** 2022-01-06

**Authors:** Yuewen Zhao, Sydney Vanderkooi, Frederick W. K. Kan

**Affiliations:** 1 Department of Biomedical and Molecular Sciences, Faculty of Health Sciences, Queen’s University, Kingston, ON K7L 3N Canada; 2 Present Address: Division of Reproductive Endocrinology and Infertility, Department of Obstetrics, Gynecology and Reproductive Sciences, Yale Fertility Center, Yale University, Orange, CT 06477 USA

**Keywords:** Oviduct-specific glycoprotein, Oviductin, OVGP1, Fertilization, Sperm-egg binding, Oviductal fluid

## Abstract

Diverse lines of evidence indicate that the mammalian oviduct makes important contributions to the complex process of reproduction other than being simply a conduit for the transport of gametes and embryos. The cumulative synthesis and transport of proteins secreted by oviductal secretory cells into the oviductal lumen create a microenvironment supporting important reproductive events, including sperm capacitation, fertilization, and early embryo development. Among the components that have been identified in the oviductal fluid is a family of glycosylated proteins known collectively as oviduct-specific glycoprotein (OVGP1) or oviductin. OVGP1 has been identified in several mammalian species, including humans. The present review summarizes the work carried out, in various mammalian species, by many research groups revealing the synthesis and secretion of OVGP1, its fate in the female reproductive tract upon secretion by the oviductal epithelium, and its role in modulating biological functions of gametes and embryos. The production and functions of recombinant human OVGP1 and recombinant OVGP1 of other mammalian species are also discussed. Some of the findings obtained with immunocytochemistry will be highlighted in the present review. It is hoped that the findings obtained from recent studies carried out with recombinant OVGP1 from various species will rekindle researchers’ interest in pursuing further the role of the oviductal microenvironment, of which OVGP1 is a major component, in contributing to the successful occurrence of early reproductive events, and the potential use of OVGP1 in improving the current assisted reproductive technology in alleviating infertility.

## Introduction

The mammalian oviductal fluid consists of a complex mixture originating from plasma transudation and glycoproteins secreted by oviduct epithelial cells (Aviles et al. 2010). The oviductal fluid in the lumen of mammalian oviducts provides a suitable milieu for gamete maturation and fertilization. Freshly ejaculated sperm are motile, but they have to undergo a series of physiological changes, collectively known as capacitation, to become fertilizing competent (Molina et al. 2018). In the in vivo condition, sperm that have reached and resided in the oviducts acquire the maximum level of fertilizing capacity. Therefore, the microenvironment of the oviductal lumen and the molecular components of oviductal fluid play an essential role in the early events of fertilization. Currently, culture media used in fertility clinics and research laboratories mimicking the physiological condition of oviductal fluid have made in vitro fertilization (IVF) possible for fertility treatments in the clinical setting, and for research purposes. Today, the global infertility rate is about 15% among couples who try to have a child but the woman fails to conceive. Infertile couples often seek assisted reproduction as a treatment. However, total fertilization failure occurs in 5–15% of couples undergoing conventional IVF despite having normal sperm quality (van der Westerlaken et al. 2005). Intracytoplasmic sperm injection (ICSI) procedure was introduced as a treatment for unexplained fertilization failure or low fertilization rate after conventional IVF (van der Westerlaken et al. 2005). ICSI is a relatively invasive procedure, and accumulating findings reported in literature have linked the association of ICSI with increased congenital disability rates compared with natural birth (Bushnik et al. 2012; Simpson 2014). Given the potential adverse effects of ICSI on the offspring, investigations into improving the success rate of conventional IVF are warranted to encourage infertile patients with mild male factor to seek the less invasive conventional IVF for treatment.

It has been suggested that oviduct-specific glycoprotein, also known as OVGP1 or oviductin, can be added as a supplement to capacitating media currently used in assisted reproductive technology (ART) procedures in fertility clinics to improve the success rate of IVF (Aviles et al. 2010). The present review provides an overview of the identification and characterization of OVGP1 and its functions, particularly its role in fertilization and early embryo development in various mammalian species, including humans. At the end of the review, the production of recombinant human OVGP1 and other recombinant OVGP1s as well as their functions are also discussed. Among the various techniques that were employed to study the properties and functions of OVGP1, immunocytochemistry at both the light and electron microscopic levels has been an important investigative tool used in conjunction with other techniques in contributing to our knowledge and understanding of the synthesis, secretion, sites of localization, and functions of mammalian OVGP1. Some of the findings obtained with immunocytochemistry will be highlighted in the present review.

## Prologue

**Fig. 1:**
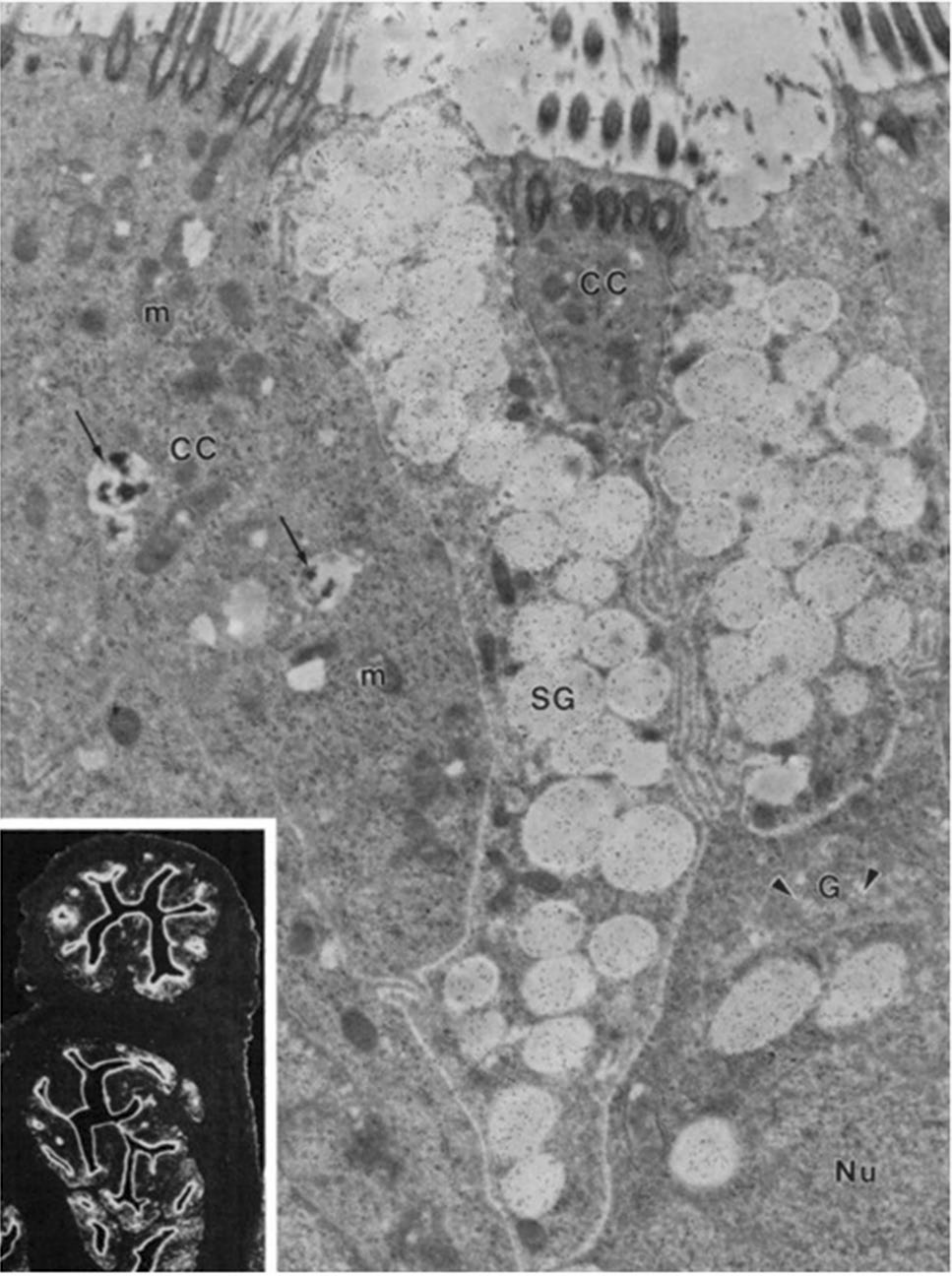
Electron photomicrograph showing the hamster oviduct epithelial cells at the ultrastructural level. Labeling for OVGP1, indicated by gold particles, is dense over the secretory granules (SG) of the nonciliated, secretory cells. Gold particles are also seen over the Golgi apparatus (G) and its associated vesicles (arrowheads). Ciliated cells (CC), characterized by their apical cilia and the presence of lysosome-like bodies (arrows) in the cytoplasm, are not labeled. *Nu* nucleus, *m* mitochondria. Magnification, 15,200×. Inset: Paraffin section of hamster oviductal tissue labeled with monoclonal antibody against hamster OVGP1. Immunostaining is limited to the epithelial cells. Magnification, 200×. (From Kan et al. 1989)

The mammalian oviduct occupies a strategic site in the female reproductive tract where fertilization takes place. The oviduct provides a suitable milieu in its lumen for many early events of reproduction to take place. The oviduct secretes many proteins, among which is a family of highly glycosylated protein named oviduct-specific glycoprotein, also known as OVGP1 or oviductin, which is exclusively synthesized and secreted by nonciliated secretory cells in the oviductal epithelium (Fig. 1). Since the discovery in hamster that the oviduct-derived glycoprotein was able to bind to the zona pellucida of ovulated hamster oocytes (Fox and Shivers 1975), the glycoprotein has been further characterized in hamster (Leveille et al. 1987) and identified and studied in other species, including mice (Kapur and Johnson 1985, 1986), rabbits (Oliphant and Ross 1982; Oliphant et al. 1984), dogs (Saint-Dizier et al. 2014), cats (Hachen et al. 2012a), sheep (Sutton et al. 1984; 1986; Gandolfi et al. 1989), pigs (Buhi et al. 1990), cows (Malayer et al. 1988; Boice et al. 1990a, b), rhesus monkeys (Verhage et al. 1997), baboons (Fazleabas and Verhage 1986; Verhage et al. 1989, 1990), and humans (Verhage et al. 1988). Except for the mouse, mammalian species examined to date showed the binding of OVGP1 to the zona pellucida of post-ovulatory oocytes of the same species. A proteomic study done in sheep indicated that OVGP1 is the most abundant protein found in the oviductal fluid with a 1.7–5.5 fold increase during the estrus stage compared with the diestrus stage (Soleilhavoup et al. 2016).

Accumulating evidence indicates that mammalian OVGP1 exerts positive effects during early events of fertilization and early embryo development. However, the molecular mechanisms that regulate the physiological function of OVGP1 in these events are not fully understood. This is mainly due to the fact that it is costly and technical difficult to obtain large quantities of oviductal fluid for subsequent purification of OVGP1 in rodents and farm animals. In humans, it is ethically impossible to obtain adequate amounts of oviductal fluid for purification of OVGP1 for functional and mechanistic studies. In our laboratory, we successfully produced recombinant hamster OVGP1 (rHamOVGP1) using recombinant DNA technology to study the role of OVGP1 in early events of fertilization including sperm capacitation, acrosome reaction, and sperm binding to zona pellucida (ZP) in vitro (Yang et al. 2015). Subsequently, we produced recombinant human OVGP1 (rHuOVGP1) to examine the role of human OVGP1 in enhancing human sperm functions (Yang et al. 2012; Zhao et al. 2016; Zhao and Kan 2019). Details of the identification and functional properties of various OVGP1s identified and the production of recombinant OVGP1s are described below.

## Molecular characterization of OVGP1

OVGP1 identified earlier in humans (Arias et al. 1994), baboons (Donnelly et al. 1991), rhesus monkeys (Verhage et al. 1997), hamsters (Suzuki et al. 1995), mice (Sendai et al. 1995), cows (Sendai et al. 1994), pigs (Buhi et al. 1996), and sheep (Desouza and Murray 1995) was found to be very conserved among species (Buhi 2002). Subsequently, OVGP1 cDNA sequence was also determined in rabbits (Yong et al. 2002) goats (Pradeep et al. 2011), and cats (Hachen et al. 2012a). Sequence analysis indicated that many of these OVGP1s have a high degree of identity (77–84%) and similarity (86–90%) in the N-terminal region of the protein core and a low degree of identity (37–63%) and similarity (50–75%) in the C-terminal region (Buhi 2002). The mature and fully glycosylated OVGP1s vary in molecular weight (90–95 kDa in domestic animals; 110–150 kDa in primates; 160–350 kDa in rodents). However, the size of the protein core of several species studied to date has been found to be similar with a molecular weight of approximately 70 kDa. The variability in molecular weight is attributed to differences in glycosylation patterns (Roux et al. 1997; Verhage et al. 1997). For example, whereas the protein core of hamster OVGP1 has a molecular weight of 71 kDa, OVGP1 purified from hamster oviductal secretion is a mucin-type glycoprotein with a molecular weight of 160–350 kDa (Robitaille et al. 1988; St-Jacques and Bleau 1988; Paquette et al. 1995). The protein core of human OVGP1 is 75 kDa, whereas the secreted glycosylated form is about 110–130 kDa (O’Day-Bowman et al. 1995).

**Fig. 2:**
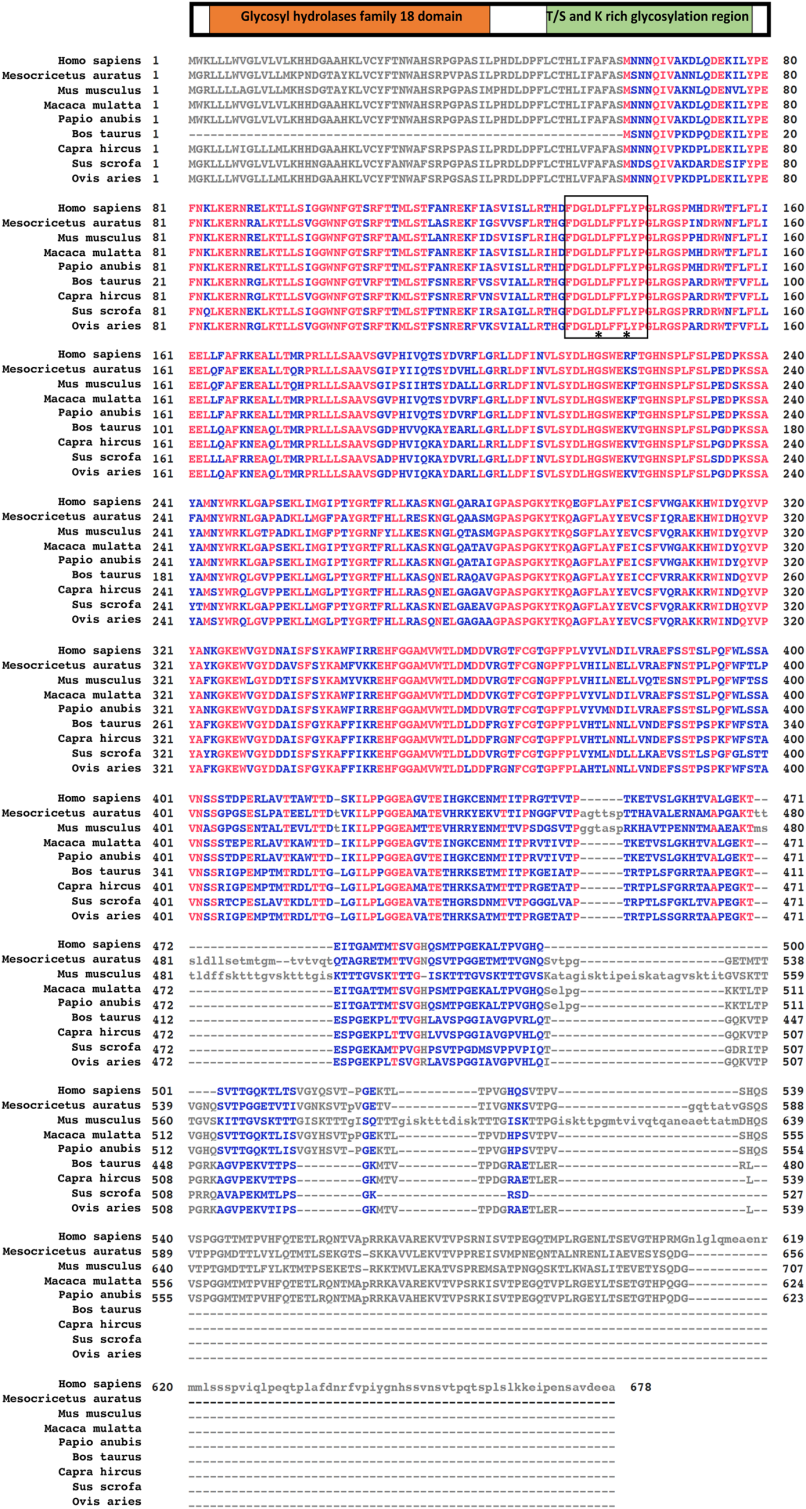
Chart showing the multiple protein sequence alignment of OVGP1 in nine different mammalian species. The top horizontal schematic shows two main regions of the OVGP1 with the N-terminal conserved glycosyl hydrolase family 18 (GH18 or chitinase) domain and C-terminal variable glycosylation region. The main schematic below shows the alignment of OVGP1 amino acid sequences of human (*Homo* *sapiens*, accession number: AAI36407.1), hamster (*Mesocricetus* *auratus*, NP_001268266.1), mouse (*Mus* *musculus*, AAI37996.1), monkey (*Macaca* *mulatta*, NP_001036252), baboon (*Papio* *anubis*, NP_001106087), cow (*Bos* *taurus*, NP_001073685), goat (*Capra* *hircus*, ABF20534), pig (*Sus* *scrofa*, NP_999235), and sheep (*Ovis* *aries*, NP_001009779) using constraint-based multiple alignment tool. The red-colored sequences are identical sequences, and the blue-colored sequences are less conserved sequences. Gray color indicates the columns that contain gaps. Where less than 50% of the sequences contain gaps, they are shown in gray uppercase and, where greater than 50% contain gaps, in gray lowercase. The black box indicates where the predicted enzymatic sites of chitinase locate on the OVGP1. Glu and Asp residues essential for the glycohydrolytic activity of the chitinase are marked by an asterisk (*)

The protein sequence alignment of OVGP1 in human, hamster, mouse, monkey, baboon, cow, goat, pig, and sheep OVGP1 is shown in Fig. 2. In silico motif scanning of OVGP1 protein revealed a domain that resembles the glycoside hydrolase 18 (GH18) family of chitinases in the N-terminal region (Malette et al. 1995b). However, the catalytic domain of OVGP1 lacks an essential glutamic acid residue that donates a proton for the hydrolysis reaction, making OVGP1 an inactive chitinase (Huang et al. 2012). Hydrophobic cluster analysis indicated that amino acids 386–525 in pig OVGP1 correspond to a C-terminal chitin-binding domain (Buhi et al. 1996). Exon arrangement analysis also revealed the chitin-binding domain in the C-terminal region of human and mouse OVGP1 (Huang et al. 2012). Due to the divergence in the protein sequence of the C-terminal domain of OVGP1, this region is likely to constitute both a binding domain and a species-specific recognition domain (Buhi 2002). Studies aiming to decipher the functional domains in OVGP1 may give a better understanding of the molecular mechanism of OVGP1.

The presence of mucin-type variable number tandem-repeat (VNTR) in the C-terminal region of OVGP1 also classifies OVGP1 as a member of the mucin family (Lapensée et al. 1997). The C-terminal region of hamster OVGP1 contains six VNTR sequences with 15 amino acids in each repeat (Paquette et al. 1995), while a similar region in the mouse OVGP1 contains 21 repeat sequences, each composed of seven amino acids (Sendai et al. 1995). In humans, baboons, and rhesus monkeys, the C-terminal region of OVGP1 contains only four tandem repeats, whereas cattle, sheep, and pigs have incomplete or no tandem-repeat sequences in the C-terminal region (Buhi 2002). Considering that these VNTR regions are usually heavily *O*-glycosylated, hamster and mouse OVGP1 that have more VNTR tandem repeats may be more closely related to mucins than their counterparts in other species. In addition to *O*-linked glycosylation sites, *N-*glycosylation sites also exist in the carboxy-terminal region. For example, human OVGP has four potential *N*-linked glycosylation sites near the C terminus (Malette et al. 1995b). Unlike the other classical members of the mucin family, OVGP1 is a secreted non-gel-forming unclassified mucin, suggesting different functional roles of this glycoprotein.

Glycosylation plays a critical role in the biological activity of OVGP1. The glycans of OVGP1 appear to be essential for maintaining the hydrophilicity of the protein in the extracellular environment, as complete deglycosylation of OVGP1 resulted in an insoluble protein (Satoh et al. 1995). Removal of sialic acid or *N*-linked glycans from bovine OVGP1 significantly reduced the ability of bovine OVGP1 to maintain sperm viability (Satoh et al. 1995). Furthermore, the addition of human OVGP1 inhibited hamster sperm binding to hamster oocytes, while the binding of hamster sperm to homologous oocytes was increased in the presence of hamster OVGP1 (Schmidt et al. 1997). Similarly, baboon OVGP1 inhibited the binding of human sperm to human zona pellucida, whereas human OVGP1 enhanced it as baboon OVGP1 shares 95% sequence similarity with human (O’Day-Bowman et al. 1996). Differences in tandem repeats, the presence and distribution of *N-* and *O*-linked carbohydrate chains, the diversity of side chains, and the length of the C-terminal region may confer species specificity, mediate specific recognition events, and regulate the biological activity of OVGP1.

The expression and secretion of OVGP1 in the oviductal epithelium are differentially regulated during the menstrual cycle or estrous cycle in mammals. An incomplete estrogen-responsive element (iERE) (5′-GGTCANNNTGACT-3′) was found at the position 150–162 bp before the start codon of the promoter region of human OVGP1 (HuOVGP1) gene (Agarwal et al. 2002). DNA sequence analysis of the human OVGP1 putative promoter revealed little homology with its hamster and mouse OVGP1 gene counterparts. However, the same iERE site was found in the promoter region of OVGP1 in hamsters and mice. The iERE sequence of HuOVGP1 is capable of binding to estrogen receptor ERβ but not ERα. Evidence of estrogen-regulated OVGP1 expression was revealed in several species, including human (Verhage et al. 1988; Arias et al. 1994; O’Day-Bowman et al. 1995; Lok et al. 2002), baboon (Verhage et al. 1990; Arias et al. 1994; Jaffe et al. 1996), sheep (Sutton et al. 1984; Buhi et al. 1991), cow (Malayer et al. 1988; Boice et al. 1990a, b), pig (Buhi et al. 1990, 1996), and hamster (McBride et al. 2004a). In species with long ovulatory cycles, the maximum mRNA and protein expression levels of OVGP1 have been found during the late follicular phase of the menstrual cycle in humans (Arias et al. 1994; O’Day-Bowman et al. 1995; Lok et al. 2002) or during the first two days of the estrous cycle in pigs (Buhi et al. 1996). OVGP1 mRNA and protein expression levels are very low or absent during the luteal phase and diestrus stage (Buhi et al. 1991, 1996; Arias et al. 1994; O’Day-Bowman et al. 1995; Verhage et al. 1998; Lok et al. 2002). Hormone replacement with estrogen after ovariectomy can restore the levels of OVGP1 mRNA and protein expression. Conversely, treatment with progesterone suppresses both mRNA and protein expression levels of OVGP1 (Buhi 2002). In hamster, the levels of OVGP1 mRNA expression are relatively the same throughout the estrous cycle (Komiya et al. 1996); however, a cyclic variation in glycosylated OVGP1 concentrations in the hamster oviduct epithelium has been found, with the highest level at the estrus stage and the lowest at diestrus (McBride et al. 2004a). A corresponding increase in glycosyltransferase activity was also found in the hamster oviduct at the time of ovulation, suggesting that glycosylation of OVGP1 may be necessary for its full functions during fertilization (McBride et al. 2004a). Based on studies carried out in various species, it appears that the protein expression of OVGP1 is under the influence of estrogen, whereas the mode of regulation and post-translational modification of OVGP1 can be different among species.

## Localization and biological functions of mammalian OVGP1

**Fig. 3:**
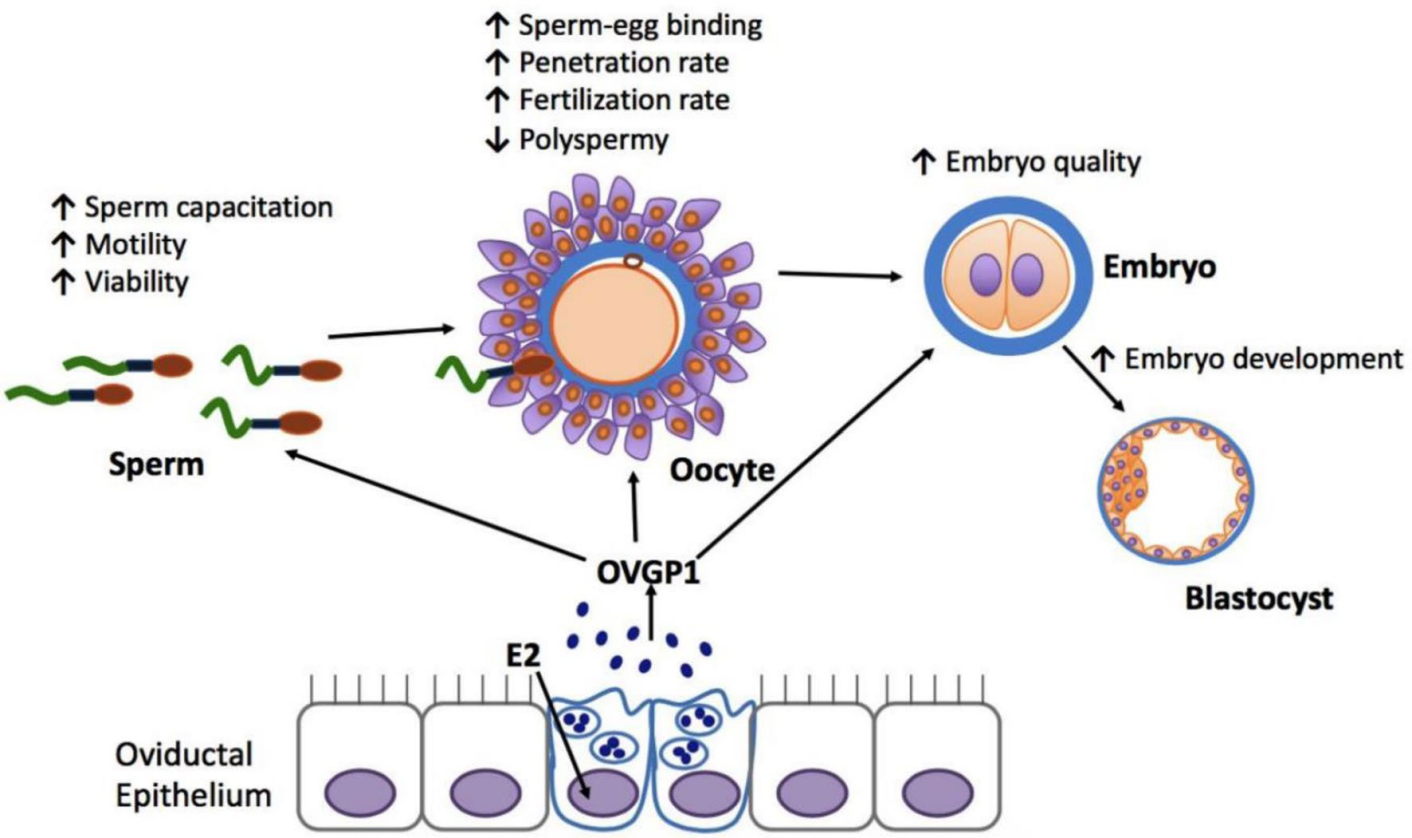
Schematic diagram summarizing the proposed biological functions of OVGP1. The synthesis and secretion of OVGP1 are influenced by the level of estrogen (E2). In vitro studies from various mammalian species have shown that native OVGP1 can bind to sperm and oocytes and enhance sperm capacitation and increase sperm motility and viability. Native OVGP1 can also enhance sperm–egg binding, increase sperm penetration rate and fertilization rate, decrease polyspermy, enhance embryo quality, and increase the number of embryos that develop to blastocysts

The concurrence of peri-ovulatory phase and high levels of OVGP1 expression suggests that this glycoprotein may play a role during early events of fertilization. The biological functions of OVGP1 during early events of fertilization that have been proposed to date are summarized in Fig. 3 and described in detail in the following sections. Briefly, in vitro studies carried out in various mammalian species have shown that native OVGP1 can bind to sperm and oocytes and exert positive effects on both sperm and oocytes, including sperm capacitation, sperm motility and viability, sperm–egg binding, penetration rate and fertilization rate, decrease in polyspermy, embryo quality, and early embryo development.

## Localization of OVGP1 in the oviductal epithelium

**Fig. 4:**
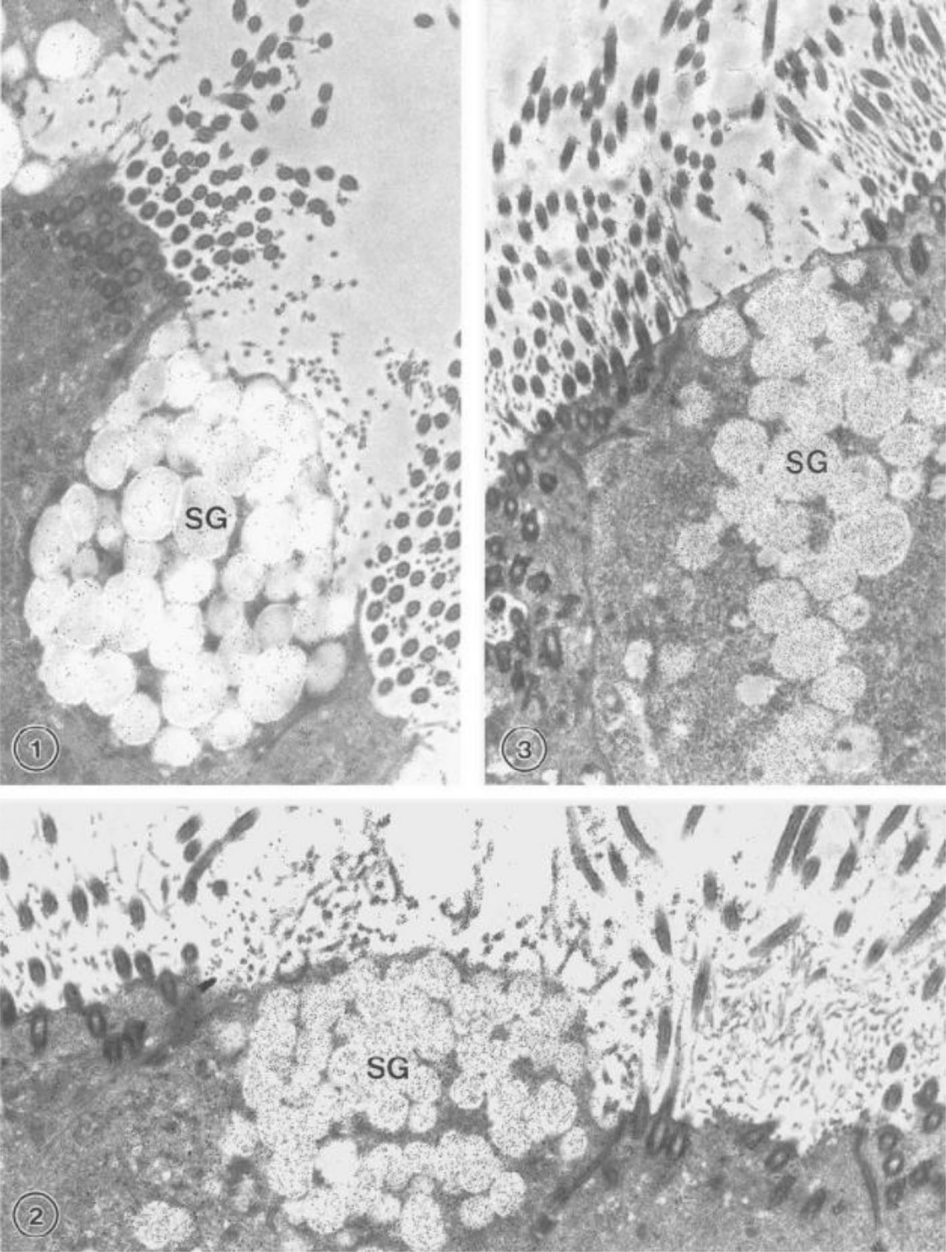
Electron photomicrographs of ultra-thin Lowicryl sections of hamster oviducts obtained from different stages of the estrous cycle and immunolabeled for hamster OVGP1 by use of the protein A-gold technique. At diestrus 1 (**1**), the secretory granules (SG) of a nonciliated secretory cell are weakly labeled by gold particles. At proestrus (**2**), the labeling density of gold particles in the secretory granules (SG) is seen to be relatively higher. The highest concentration of gold particles is found in the secretory granules (SG) of the secretory cells at estrus (**3**). Magnification, 19,000×. (From Roux and Kan 1995)

**Fig. 5:**
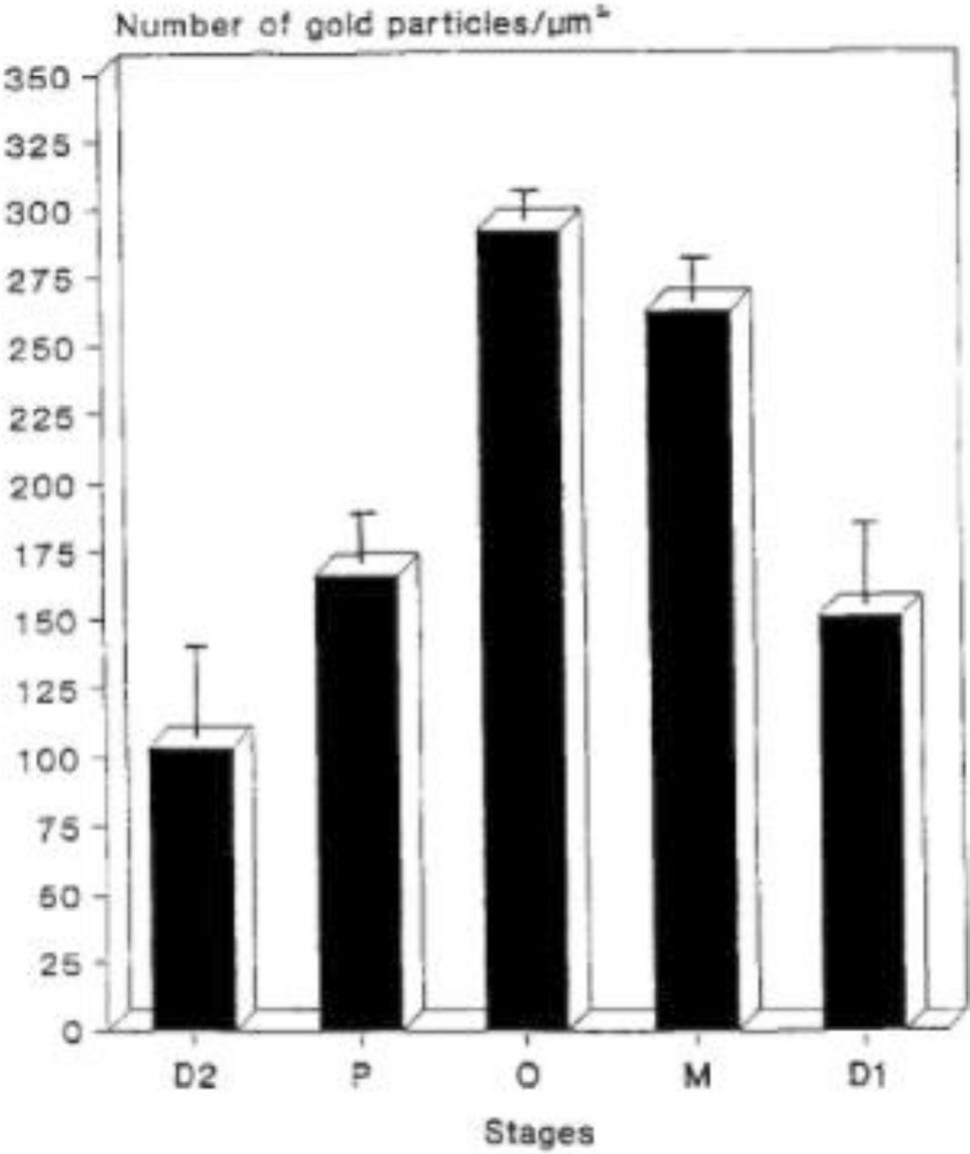
Histogram showing mean labeling density of hamster OVGP1 [expressed in histogram as the number of gold particles per µm^2^ ± standard error of the mean (SEM)] in the secretory granules of hamster oviduct obtained from five different stages of the estrous cycle. *D2* diestrus, *P* proestrus, *O* estrus, *M* metestrus, *D1* diestrus 1. (From Roux and Kan 1995)

OVGP1 is synthesized and secreted by secretory cells in the oviductal epithelium (St-Jacques and Bleau 1988; Maines-Bandiera et al. 2010; Saint-Dizier et al. 2014). Northern blot analysis showed that mRNA transcripts for OVGP1 are found exclusively in the oviduct and not in any other tissues in hamsters (Suzuki et al. 1995). The protein expression of OVGP1 gradually increases as the animal becomes sexually mature (Malette et al. 1995a). At the ultrastructural level, OVGP1 was found to be localized within the secretory granules and Golgi saccules in nonciliated secretory cells but not ciliated cells of the oviductal epithelium in hamster (Fig. 1) (Kan et al. 1989; McBride et al. 2004a). Results obtained with human oviducts using immunohistochemistry, Western blotting, and reverse-transcription polymerase chain reaction (RT-PCR) showed that maximal production of OVGP1 occurs around the time of ovulation (O’Day-Bowman et al. 1995; Lok et al. 2002). In hamster, an increased level of expression of mature and fully glycosylated OVGP1 occurs during the peri-ovulatory phase as demonstrated by quantitative immunocytochemistry (Figs. 4 and 5), suggesting an important role for OVGP1 in the fertilization process.

## Binding of OVGP1 to sperm

**Fig. 6:**
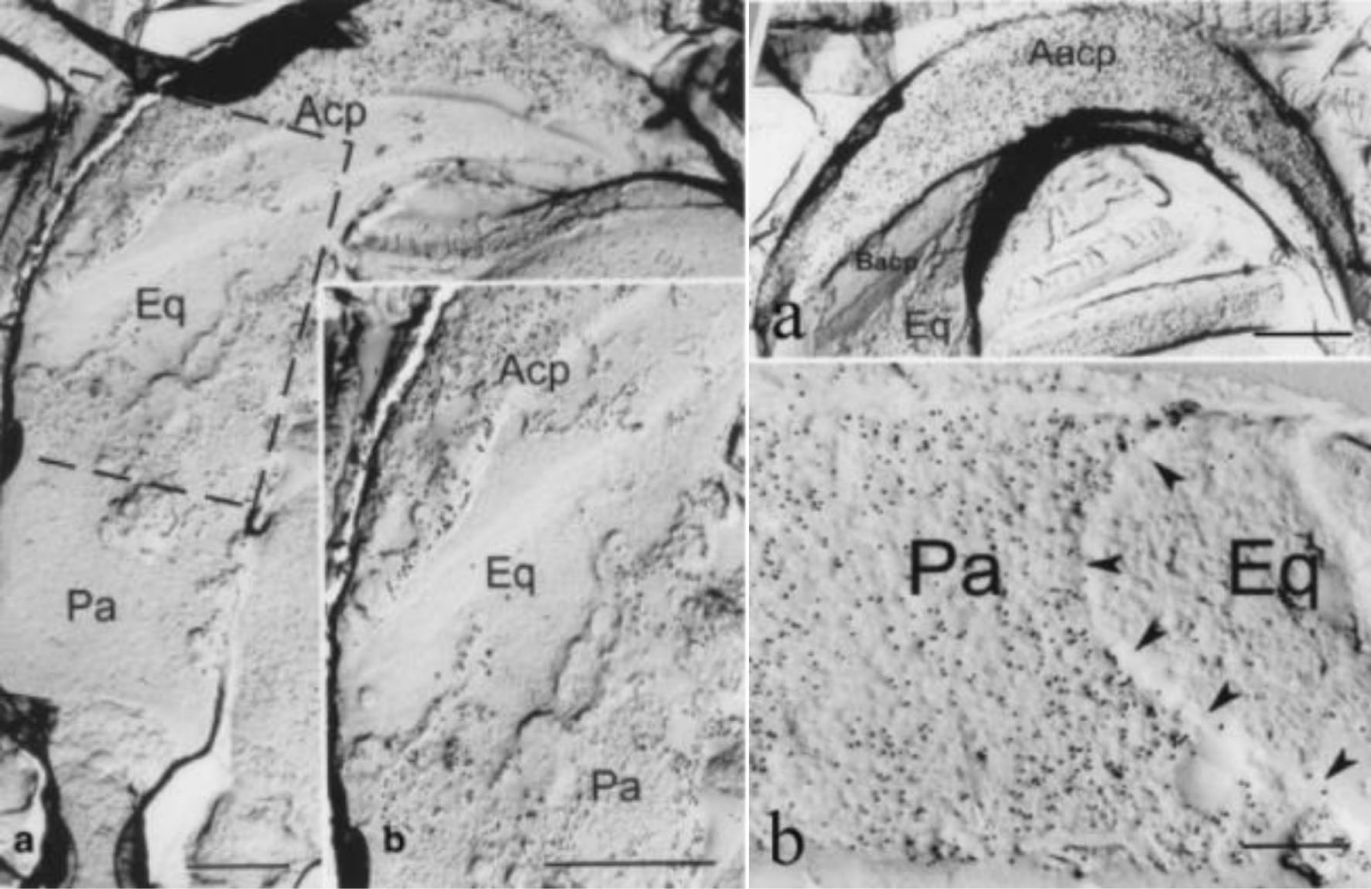
Left: surface replica preparation showing the overall distribution of immunogold labeling over the sperm head of a noncapacitated hamster sperm (**a**). *Acp* acrosomal cap region, *Eq* equatorial segment, *Pa* post-acrosomal region. Image in (**b**) represents a high magnification of the region delimited by the box in (**a**) showing a high concentration of gold particles over the acrosomal cap region (Acp). Moderate and relatively weak labeling are also detected, respectively, over the post-acrosomal region (Pa) and equatorial segment (Eq). **a** and **b**: Bars, 1.0 µm. Right: surface replicas showing increased labeling by gold particles in specific regions of the sperm head after capacitation. Image in (**a**) shows the more intensely labeled apical acrosomal cap region (Aacp) as compared with the basal acrosomal cap region (Bacp). In (**b**), the post-acrosomal region (Pa) is also strongly labeled by gold particles while only a few gold particles are seen over the equatorial segment (Eq). The post-acrosomal region and equatorial segment are separated from each other by the posterior border of the equatorial segment (arrowheads). **a**: Bar, 0.1 µm; **b**: Bar, 0.5 µm. (From Kan and Esperanzate 2006)

OVGP1 has been shown to bind to sperm and has positive effects on sperm capacitation, viability, motility, and acrosome reaction (King et al. 1994; Abe et al. 1995; Killian 2004). Binding of partially purified hamster OVGP1 to the head region of homologous sperm has been reported, and the pattern of binding showed regional and temporal modifications during sperm capacitation (Boatman and Magnoni 1995). In noncapacitated sperm, OVGP1 binds to the acrosomal region of sperm head. After capacitation, OVGP1 was found to be over the equatorial segment and post-acrosomal region (Boatman and Magnoni 1995). The increase in binding of hamster OVGP1 to the acrosomal and post-acrosomal regions of homologous sperm during capacitation has been shown by immunolabeling using the surface-replica technique (Fig. 6). As OVGP1 is the major glycoprotein secreted by the oviductal epithelium, binding of OVGP1 to the head region of sperm is thought to be involved in sperm–oviduct interaction. Localization of OVGP1 to different membrane domains of mammalian sperm varies among species. In bovine, OVGP1 binds to the posterior region of the sperm head and the mid-piece of sperm tail (Abe et al. 1995). In humans, an earlier study detected the binding of oviduct-specific glycoprotein over the surface of the head region (Lippes and Wagh 1989). As described below, the use of recombinant human OVGP1 revealed its localization to specific membrane domains of human sperm.

The binding of OVGP1 to sperm appears to exert positive effects on sperm functions. Incubating bovine spermatozoa with purified OVGP1 in conditioned medium can enhance sperm capacitation, increase the potential of sperm to undergo acrosome reaction, enhance the ability of sperm to fertilize bovine oocytes (King et al. 1994; Satoh et al. 1995), and increase sperm motility and viability in a dose-dependent manner (Abe et al. 1995). Partially purified porcine OVGP1 added to the incubating medium at low concentrations was able to increase viability of porcine sperm (McCauley et al. 2003). Purified hamster OVGP1 from the estrus stage was shown to increase the levels of tyrosine phosphorylation of sperm proteins, a biochemical hallmark of sperm capacitation, in a time-dependent manner (Saccary et al*.*, 2013b). Taken together, the above findings obtained from in vitro functional studies of OVGP1 indicate the beneficial effects of the presence of OVGP1 in the capacitating medium in terms of sperm functions, suggesting that supplementing the incubating medium with OVGP1 could enhance the fertilizing competence of sperm.

## Binding of OVGP1 to oocytes

Localization of OVGP1 to the zona pellucida of oviductal oocytes was one of the characteristic features of this glycoprotein (Araki et al. 1987, O'Day-Bowman et al. 1996; Coy et al. 2008). OVGP1 is absent in the ovarian follicles in the ovary and is added to the zona pellucida of post-ovulatory oocytes during oviductal transit (Araki et al. 1987; St-Jacques and Bleau 1988; Kan et al. 1989; Abe and Oikawa 1990). The glycoprotein is specifically associated with dense filamentous structures comprising the zona matrix (Kan et al. 1990; McBride et al. 2004b). Immunolocalization studies at the ultrastructural level revealed the presence of the glycoprotein on the microvilli and in the perivitelline space of fertilized hamster oocytes and in the multivesicular bodies of early embryos (Kan et al. 1990, 1993; Kan and Roux 1995; McBride et al. 2004b). Similarly, in baboons, incubating oocytes with extracted oviductal fluid or purified homologous OVGP1 also showed binding of OVGP1 to the zona pellucida (O’Day-Bowman et al. 2002).

Binding of OVGP1 to the zona pellucida appears to modify the functions of zona pellucida. Co-incubating human sperm and hemizonae in the presence of human OVGP1 and co-incubation of hamster sperm and retrieved ovarian oocytes in the presence of hamster OVGP1 enhanced sperm–zona binding and sperm–egg penetration (Boatman and Magnoni 1995; O’Day-Bowman et al. 1996). Despite the high homology between human and baboon OVGP1, addition of baboon OVGP1 in the human hemizona binding assay resulted in a decrease in binding of sperm to hemizonae, indicating the importance of species specificity in sperm–egg binding (O’Day-Bowman et al. 1996).

Treatment of bovine and porcine ovarian oocytes with homologous OVGP1 prior to incubation with the respective sperm has shown positive effects on fertilization. Bovine oocytes preincubated with homologous OVGP1 resulted in a significant increase in fertilization rates, even with a smaller number of sperm used to fertilize the oocytes (Martus et al. 1998). In livestock, polyspermy is a common phenomenon that affects the success rate of in vitro fertilization (Suzuki et al. 2003). In pigs, polyspermy was significantly reduced when oocytes were pretreated with low concentrations of OVGP1 while high penetration and fertilization rates were maintained (McCauley et al. 2003). Similarly, in another study, porcine OVGP1 added in the IVF medium at low concentration (< 20 μg/mL) maintained high penetration rates with a significant decrease in polyspermy (Kouba et al. 2000). On the contrary, treating porcine sperm with high concentrations of OVGP1 reduced sperm penetration and binding of sperm to oocytes. Goat OVGP1 was found to be capable of increasing the resistance of zona pellucida to pronase digestion in a dose-dependent manner; however, the presence of low concentrations of OVGP1 in the incubating medium resulted in a higher cleavage rate of the fertilized oocytes, and more embryos developed to morula and blastocyst stages (Pradeep et al. 2011). Taken together, in vitro functional studies showed that OVGP1 in various mammalian species exerts positive effects on both sperm and oocytes, albeit with varying degrees among different species.

## Uptake and internalization of OVGP1 by early embryos

Uptake and internalization of OVGP1 by oocytes have been reported in the hamster and baboon, suggesting that OVGP1 may play a role in early embryo development (Kan and Roux 1995; Boice et al. 1990a, b). Following fertilization of hamster oocytes, shedding of zona pellucida as flocculent material containing OVGP1 was found in the perivitelline space of early embryos. This flocculent material was also found in coated pits, coated vesicles, multivesicular bodies, and lysosome-like structures in the blastomeres of developing embryos, indicating that OVGP1 is endocytosed and internalized by blastomeres during early embryo development (Kan and Roux 1995). Incubation of ovine spermatozoa and oocytes with OVGP1 during fertilization showed a significant increase in cleavage rate and the number of embryos developed to blastocysts (Hill et al. 1996a); however, the addition of OVGP1 to the culture medium after in vitro fertilization showed no effect on cleavage rates (Hill et al. 1996b). During in vitro fertilization in the pig in the presence of porcine OVGP1, an increase in the number of embryos that developed to the blastocyst stage was observed, although no effects on cleavage rates were noted (Kouba et al. 2000). Similarly, in sheep, while the addition of OVGP1 in the culture medium during in vitro fertilization was found to have no effects on cleavage rates, a high yield of blastocysts and a shorter time for fertilized oocytes to develop to blastocyst stage were observed (Hill et al. 1997). Therefore, results obtained from the above studies indicate that the uptake of OVGP1 by early embryos has an embryotrophic role during early embryo development.

## Extra-oviductal localization of OVGP1

Although studies of synthesis and secretion of OVGP1 indicated that OVGP1 is exclusively synthesized and secreted by the oviductal epithelium (Buhi 2002; Aviles et al. 2010), OVGP1 has been detected at the apical surface of the epithelium lining the endometrium in hamsters. Upon release from the oviductal secretory cells, hamster OVGP1 gains access to the uterine lumen and binds to the microvilli of endometrial epithelial cells and becomes internalized (Martoglio and Kan 1996; Roux et al. 1997; McBride et al. 2004b). During early gestation in hamsters, immunolabeling of OVGP1 over the cell surface of the uterine epithelium was found to decrease in intensity from day 1 to day 6 (Roux et al. 1997). In mice, the intensity of immunostaining of OVGP1 expression at the uterine epithelium was found to gradually increase following conception (Laheri et al. 2018). The level of expression was most evident on day 5 after implantation and subsequently decreased. The alteration of OVGP1 protein expression levels at the uterine epithelium during implantation suggests a possible role of OVGP1 in regulating uterine receptivity. The latter authors further used OVGP1 knockdown experiments and real-time PCR to demonstrate that loss of OVGP1 in human endometrial epithelial cells altered the expression of endometrial receptivity-related genes and reduced the in vitro adhesiveness of trophoblast cells (Laheri et al. 2018). More importantly, the authors found that expression of OVGP1 mRNA was significantly lowered in the endometrium of women with recurrent implantation failure (Laheri et al. 2018). Therefore, the above findings obtained by Laheri and coworkers (2018) reinforce the notion that OVGP1 may play a role in regulating uterine receptivity during implantation and early gestation.

While studies carried out to date in various mammalian species, including humans, indicate that the synthesis and secretion of OVGP1 is oviduct-specific, it is worth mentioning that OVGP1 has been detected in the cervix of rabbit (Hendrix et al. 2001) and rhesus macaque (Slayden et al. 2018). However, the former study carried out in the rabbit utilized mainly high-performance liquid chromatography (HPLC) and Northern blot analyses, and the latter study in rhesus macaque employed an antibody against the peptide of OVGP1. Further studies are necessary to confirm both gene and protein expression of OVGP1 in the cervix and elucidate the role of OVGP1 in this segment of the uterus.

Although OVGP1 protein expression is absent in normal human ovarian tissues (McBride et al. 2004b; Maines-Bandiera et al. 2010; Wu et al. 2016), an increased level of OVGP1 has been detected in benign ovarian tumor fluids (Poersch et al. 2016), and also in borderline tumors, stage I and II serous carcinomas, and mucinous carcinomas (Woo et al. 2004) as compared with late-stage ovarian carcinomas. The expression profile of OVGP1 in these tumors highlights the potential of using OVGP1 as a biomarker for the detection of early stages of ovarian tumorigenesis and to improve ovarian cancer diagnosis. More work needs to be done to explore the use of OVGP1 as a biomarker for early detection of ovarian cancer.

To study the physiological significance of this glycoprotein, Araki and colleagues produced *Ovgp1-*null mice (Araki et al. 2003) and showed that *Ovgp1-*null mice displayed sub-fertility phenotype (i.e., smaller litter size from the *Ovgp1-*null females and reduced ability of sperm to fertilize *Ovgp1*-null oocytes). Therefore, although OVGP1 may not be essential for in vivo reproduction in mice, its absence may result in reproductive deficits. It is also worth mentioning that, in contrast to OVGP1 identified in other mammalian species, including humans, where OVGP1 is localized to the zona pellucida, mouse OVGP1 is found in the perivitelline space of post-ovulatory oocytes instead (Kapur and Johnson 1986).

## Production of recombinant OVGP1

Further studies of the functions of mammalian OVGP1 and the mechanism that regulates its functions had been hampered by the limited amounts of native OVGP1 that can be isolated and purified from rodents and farm animals. This limitation is more apparent in studies with human OVGP1 owing to ethical reasons. In the past few years, recombinant DNA technology has been used to produce recombinant OVGP1 in several mammalian species, including hamster (Yang et al. 2015), human (Zhao et al. 2016), porcine (Algarra et al. 2016), feline (Hribal et al. 2014), and buffalo (Choudhary et al. 2017) from cell cultures to generate adequate amounts of OVGP1 for functional and mechanistic studies.

## Recombinant hamster OVGP1 (rHamOVGP1)

Our laboratory has successfully produced rHamOVGP1 in transfected HEK293 cells. Particularly, rHamOVGP1 can be purified using HPA-agarose affinity columns as hamster OVGP1 contains terminal α-d-GalNAc residues (Malette and Bleau 1993). The resulting rHamOVGP1 displays a polydispersed band of 160–350 kDa on sodium dodecyl sulfate–polyacrylamide gel electrophoresis (SDS-PAGE) gel, with its true identity confirmed by proteomic analysis (Yang et al. 2015). The molecular mass of rHamOVGP1 corresponds to that of the purified native protein previously described (Malette and Bleau 1993). Similar to native HamOVGP1 (Boatman & Magnoni 1995; Kan & Esperanzate 2006; Saccary et al. 2013), rHamOVGP1 binds to homologous sperm, specifically to the head and mid-piece of hamster epididymal sperm and throughout the entire thickness of the zona pellucida of oocytes, suggesting that rHamOVGP1 may exert its effects through its binding to the head and tail regions of hamster sperm (Yang et al. 2015). Native HamOVGP1 has previously been shown to enhance sperm capacitation by increasing the level of tyrosine phosphorylation of a number of sperm proteins that are known to be involved in sperm-egg binding and sperm motility (Saccary et al. 2013). These tyrosine-phosphorylated proteins are associated with the sperm head and tail, respectively (Saccary et al. 2013). Similarly, addition of rHamOVGP1 to the capacitating medium further enhances sperm capacitation by increasing the levels of protein tyrosine phosphorylation in hamster sperm in a time-dependent manner (Yang et al. 2015). The rHamOVGP1-enhanced tyrosine-phosphorylated proteins are predominantly located in the sperm tail, suggesting a role of OVGP1 in enhancing sperm motility during capacitation. Sperm capacitation is thought to prepare sperm to undergo the subsequent acrosome reaction. Incubating hamster sperm with rHamOVGP1 in capacitation medium can significantly increase the percentage of acrosome-reacted sperm, thus increasing the fertilizing potential of sperm (Yang et al. 2015). rHamOVGP1 binds to the zona pellucida (ZP) of hamster oocytes throughout its entire thickness. Treatment of homologous sperm and/or oocytes with rHamOVGP1 prior to sperm–egg binding assay can significantly increase the number of sperm bound to the ZP of oocytes (Yang et al. 2015). The positive effect of rHamOVGP1 on sperm–oocyte binding appears to be more associated with the oocyte since the largest increase in the number of sperm bound to oocytes was noted when the sperm–egg binding essay was performed in the presence of rHamOVGP1 with pretreated oocytes and untreated sperm (Yang et al. 2015). Successful production of glycosylated recombinant hamster OVGP1 provides a valuable tool to study the biological function and the molecular mechanism of OVGP1 in mammalian fertilization.

## Recombinant human OVGP1 (rHuOVGP1)

Despite the findings from animal models supporting a role for OVGP1 in fertilization and early embryo development, information concerning the biological role of human OVGP1 is lagging behind its counterpart in other mammalian species. Our laboratory utilized recombinant DNA technology to produce, for the first time, the secretory form of human OVGP1 in HEK293 cells (Zhao et al. 2016). The purified rHuOVGP1 showed a single band corresponding to the 120–150 kDa size range of native human OVGP1, and its identity as human OVGP1 was further confirmed by mass spectrometric analysis (Zhao et al. 2016). HEK293 cells possess biosynthetic pathways of *N*- and *O*-glycosylation similar to those found in immortalized human oviductal cells (Yang et al. 2012). Therefore, it is reasonable to believe that glycosylation of rHuOVGP1 in HEK293 cells is very similar to that of native HuOVGP1 in human oviductal cells.

**Fig. 7:**
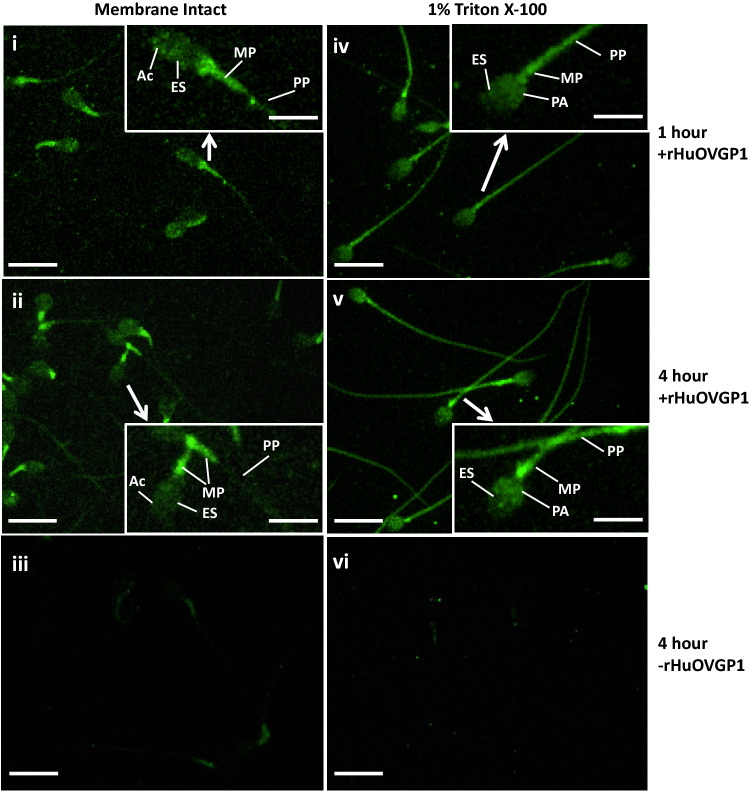
Binding of rHuOVGP1 to human sperm during in vitro capacitation. Confocal microscopy imaging of human sperm incubated in the presence of rHuOVGP1 shows binding of the recombinant glycoprotein to membrane-intact sperm (**i** and **ii**) and 1% Triton X-100-treated sperm (**iv** and **v**) following 1 h (**i** and **iv**) and 4 h (**ii** and **v**) of capacitation. Controls show negative immunostaining when sperm were incubated in the absence of rHuOVGP1 for the membrane-intact sperm (**iii**) and Triton-treated sperm (**vi**). Scale bars, 10 µm. Insets: high magnifications of sperm cells revealing various immunolabeled structures. *Ac* acrosomal region, *ES* equatorial segment, *MP* mid-piece, *PA* post-acrosomal region, *PP* principal piece. Scale bar, 5 µm. (From Zhao et al. 2016)

In membrane-intact sperm, rHuOVGP1 binds to the acrosomal region and equatorial segment of the sperm head, the connecting piece of the sperm neck, the mid-piece, and, to a lesser extent, the principal piece of the sperm tail (Fig. 7). Interestingly, when treated with 1% Triton X-100, a non-ionic surfactant that solubilizes cell membranes and peripheral membrane-bound proteins, the binding of rHuOVGP1 displayed an altered pattern. Confocal microscopy imaging revealed relatively intense immunostaining of rHuOVGP1 over the equatorial and post-acrosomal regions in the sperm head and throughout the mid-piece and principal piece of the tail (Fig. 7). The presence of rHuOVGP1 in both the Triton-soluble fraction and insoluble fraction (Zhao et al. 2016), along with the findings above, suggests the association of rHuOVGP1 not only with the plasma membrane of sperm but also with certain structural elements underlying the cell surfaces. Similar to the biologically active recombinant hamster OVGP1 (Yang et al. 2015), the presence of rHuOVGP1 in the incubating medium can significantly increase tyrosine phosphorylation of a subset of sperm proteins during capacitation in a time-dependent manner, and significantly increase the number of acrosomal-reacted sperm (Zhao et al. 2016). Although it is not clear whether the increase in acrosome reaction is directly linked to the increase in protein tyrosine phosphorylation under the influence of rHuOVGP1, the presence of rHuOVGP1 in the incubating medium can likely increase the fertilizing capacity of the sperm following capacitation.

**Fig. 8:**
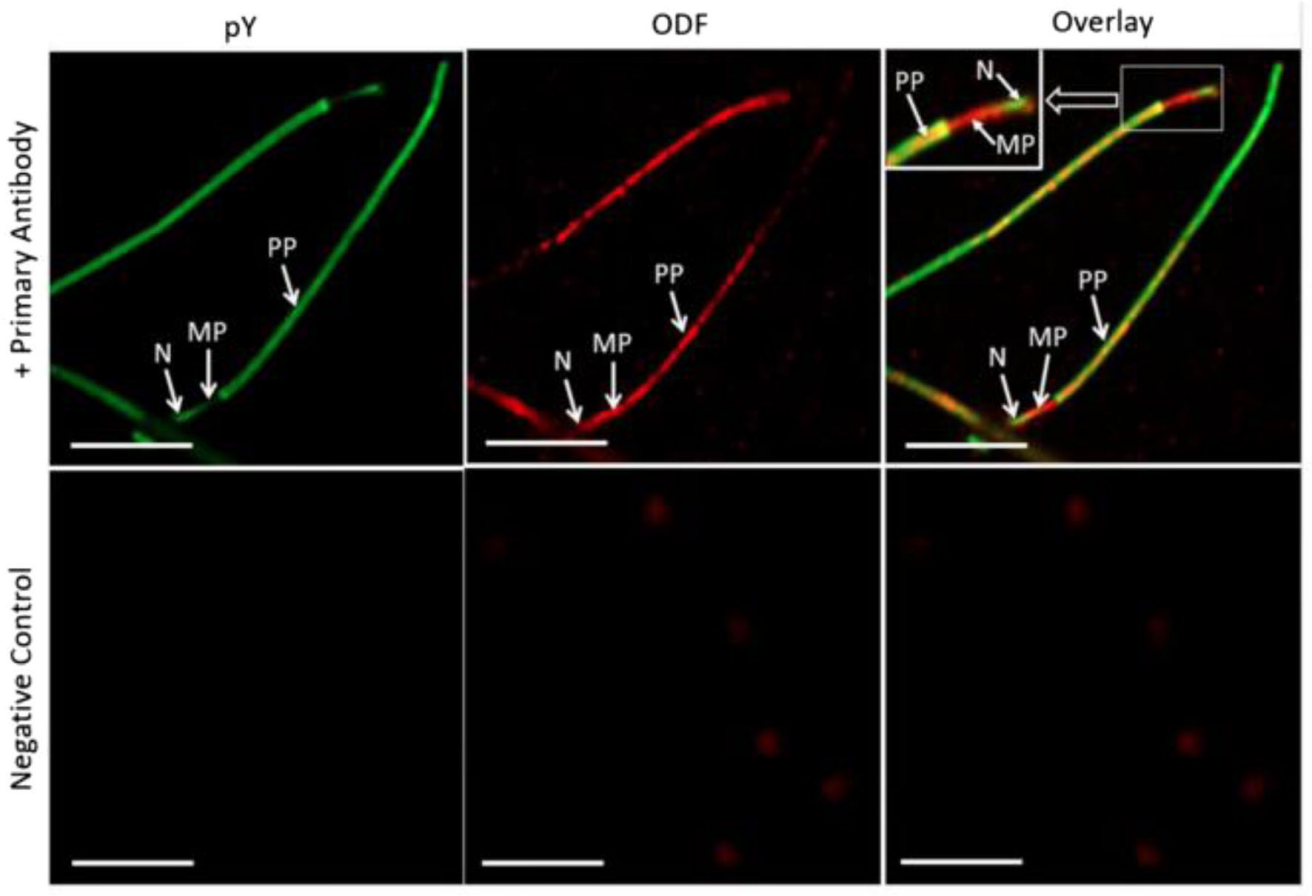
Tyrosine-phosphorylated proteins are predominantly localized to the fibrous sheath of the sperm tail. Confocal fluorescent images showing the immunofluorescent labeling of tyrosine-phosphorylated sperm proteins (pY) (upper left), the outer dense fiber (ODF) protein (upper middle), the overlay image (upper right), and corresponding negative controls (lower panels). Insets: high magnifications of sperm cells within the framed boxes revealing various immunolabeled structures. Scale bar, 10 µm. *N* neck region, *MP* mid-piece, *PP* principal piece. (From Zhao and Kan 2019)

**Fig. 9:**
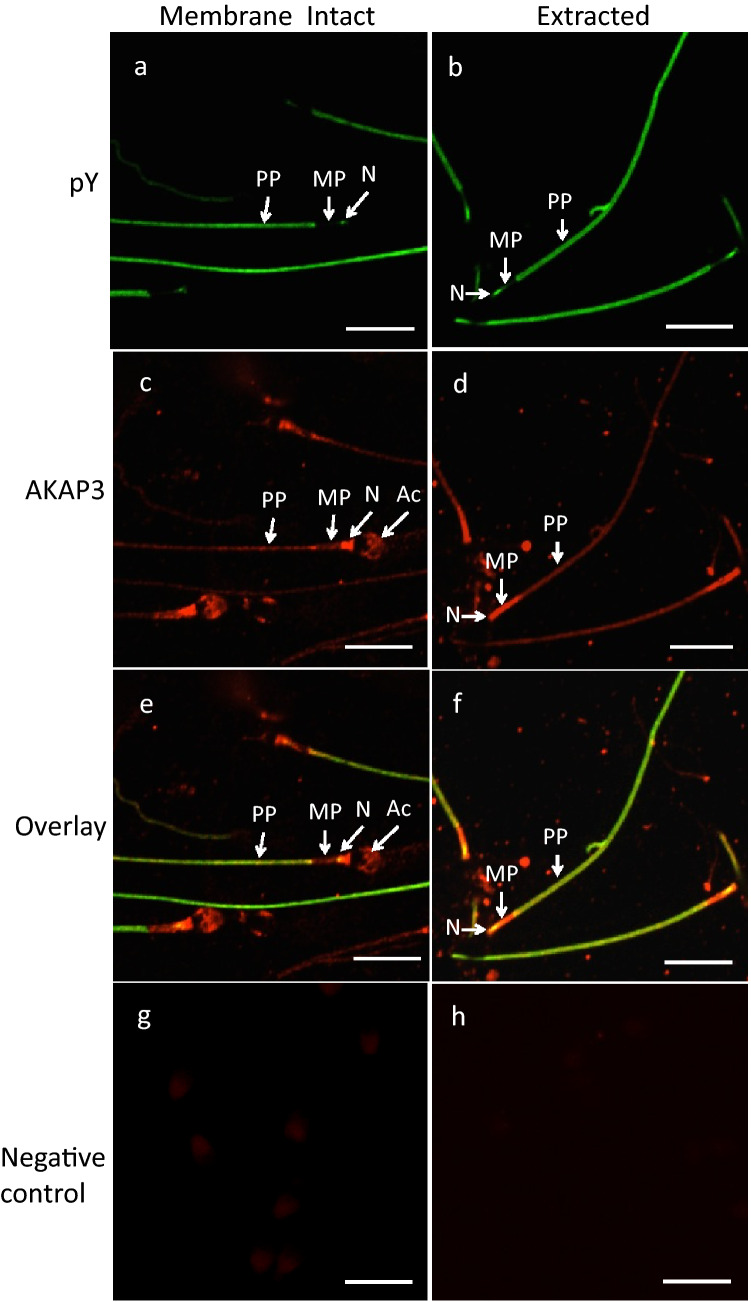
AKAP3 is co-localized with pY proteins in the neck region and in the principal piece of the sperm tail. Confocal microscopic images of immunofluorescent labeling of pY proteins (**a** and **b**) and AKAP3 (**c** and **d**) on capacitated sperm with intact membrane (**a**, **c**, and **e**) and Triton-DDT-extracted (**b**, **d**, and **f**) sperm. Bottom panel (**g** and **h**) shows the negative control of AKAP3 labeling. Insets: high magnifications of sperm cells within the framed boxes revealing various immunolabeled structures. Scale bar, 10 µm. *N* neck region, *MP* mid-piece, *PP* principal piece, *Ac* acrosome. (From Zhao and Kan 2019)

**Fig. 10:**
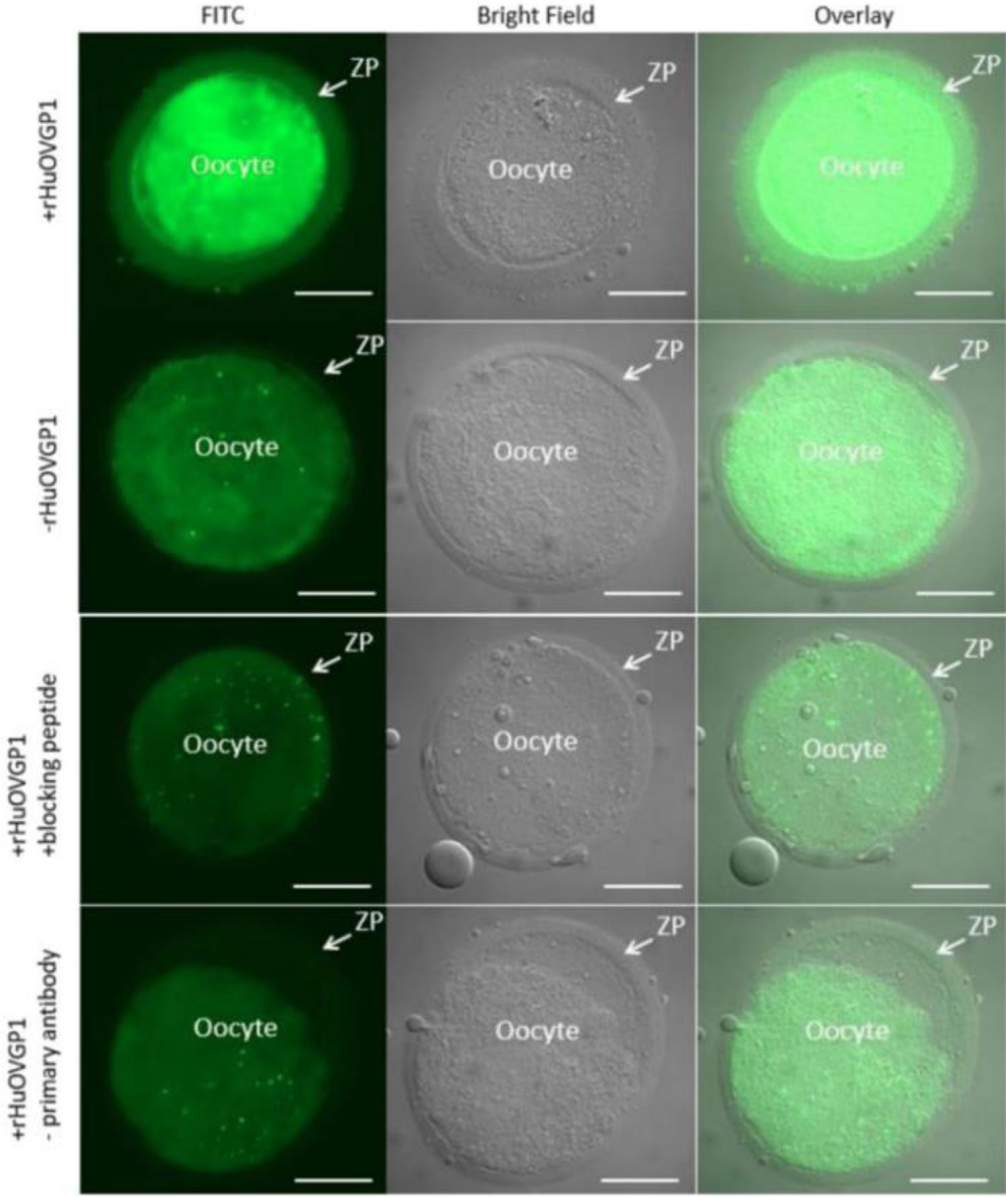
rHuOVGP1 binds to the zona pellucida (ZP) of human oocytes. Microscopic images showing the immunofluorescent labeling of rHuOVGP1 in human oocytes following incubation of oocytes in the presence (+) or absence (−) of rHuOVGP1, with the blocking peptide specific for the OVGP1 antibody, or in the absence (−) of the primary antibody during immunolabeling, respectively. Labeling in the oocyte proper is nonspecific. Scale bar, 50 µm. (From Zhao and Kan 2019)

Tyrosine-phosphorylated proteins are located predominantly in the fibrous sheath in the principal piece of the sperm tail (Fig. 8). The fibrous sheath has been suggested to influence the degree of flexibility, flagellar motion, and shape of the flagellar beat while functioning as a scaffold for proteins in signaling pathways involved in sperm motility and capacitation (Eddy et al. 2003). Two major components of the fibrous sheath are A-kinase anchoring proteins (AKAP) 3 and 4 (Eddy et al. 2003). Binding of protein kinase A (PKA) to tyrosine-phosphorylated AKAP3, the latter of which has been localized to the fibrous sheath of human sperm tail (Fig. 9), plays an important role in sperm motility (Luconi et al. 2005; Pereira et al. 2017). Sperm incubated with rHuOVGP1 can further potentiate the efficiency of sperm to undergo acrosome reaction (Zhao et al. 2016), which is linked to increased fertilization rates. Similar to recombinant hamster OVGP1, which binds to the zona pellucida (ZP) of homologous ovarian oocytes, rHuOVGP1 also binds to the ZP of human oocytes (Fig. 10). Treatment of both hemizonae and sperm with rHuOVGP1 prior to co-incubation in hemizona binding assays can increase the number of sperm bound to the ZP of oocytes as compared with similar conditions in the absence of rHuOVGP1 (Zhao and Kan 2019).

Accumulating evidence indicates that supplementation of rHuOVGP1 in capacitating medium in standard in vitro fertilization procedures could be beneficial for increasing the fertilization success rate. Today, a major limitation of human IVF therapy is failed sperm binding and lack of fertilization mainly due to male infertility indications. Supplementing the capacitating medium with rHuOVGP1 may be useful for treatment of male infertility with mild cases of male factor. Since studies with both rHamOVGP1 and rHuOVGP1 suggest that, in both cases, the recombinant glycoproteins enhance sperm capacitation, in part, through the increase in the level of tyrosine phosphorylation of sperm proteins, predominantly in the sperm tail, that are essential for sperm motility, what remains to be determined in the future is the mechanism of OVGP1 that regulates the increase in tyrosine phosphorylation of sperm proteins during capacitation and the enhancement of sperm–egg binding. A major mechanism of sperm capacitation is the influx of calcium (Ca^2+^) (Rahman et al. 2014; Jin and Yang 2017). Cumulus cells that surround the ovulated oocytes produce high concentrations of progesterone. Progesterone is known to induce Ca^2+^ influx and hyperactivation of sperm motility through the cation channels of sperm (Catsper) located in the sperm tail (Lishko et al. 2011; Miller et al. 2016). Our laboratory is currently investigating if HuOVGP1 can increase the intracellular Ca^2+^ concentrations during sperm capacitation, and, if so, studies will be carried out to examine if HuOVGP1 exerts its effect in a manner similar to that of progesterone via the Catsper channels.

## Recombinant OVGP1 in other mammalian species

In addition to the aforementioned recombinant OVGP1 of human and hamster, recombinant OVGP1 has also been produced and examined in other mammalian species. A study using recombinant porcine OVGP1 demonstrated that both full-length and truncated forms of OVGP1 were able to bind to porcine zona pellucida; however, full-length OVGP1 was able to penetrate deeper into the zona pellucida as compared with the truncated forms of recombinant porcine OVGP1 and recombinant rabbit OVGP1, and increase the efficient rate of in vitro fertilization (Algarra et al. 2016). The study also demonstrated that the C-terminal region of recombinant porcine OVGP1 is essential for regulating several functional parameters of the ZP and its endocytosis by in vitro matured oocytes (Algarra et al. 2016). A subsequent study carried out by the same group showed that recombinant porcine OVGP1 did not affect the development rates of bovine embryos during in vitro culture, but its presence during in vitro fertilization and embryonic in vitro culture was able to produce embryos that carry relatively more embryo-specific genes (Algarra et al. 2018). However, the authors in the latter study did not observe positive effects of recombinant porcine OVGP1 on the development rates of bovine embryos. This could be due to the fact that the recombinant OVGP1 used in the latter study was not from a homologous species but from pig instead of from bull. Buffalo and feline recombinant OVGP1s have been produced, respectively, by two other research groups using a bacterial expression system (Hachen et al. 2012b; Choudhary et al. 2017). Unlike the hamster, human, and porcine recombinant OVGP1s that were produced in HEK293 cells, the buffalo and feline recombinant OVGP1s are not glycosylated. Although these two groups have shown that these unglycosylated recombinant OVGP1s can also benefit sperm capacitation and the fertilization process, the potential of the enhancement was thought to be less than their respective glycosylated native proteins (Hribal et al. 2014; Choudhary et al. 2017). This further implicates the glycans as important functional components of OVGP1 in mammals.

## Conclusion

The present review summarizes the major findings of studies carried out in various mammalian species regarding the role of oviduct-specific glycoprotein, also known as oviductin or OVGP1, in regulating the biological functions of gametes and early embryos. Collectively, in vitro functional studies suggest that OVGP1 plays a key role in sperm capacitation, fertilization, and early embryo development. Of particular interest is the use of both recombinant hamster OVGP1 (rHamOVGP1) and recombinant human OVGP1 (rHuOVGP1) in demonstrating the association of OVGP1-enhanced tyrosine phosphorylation of sperm proteins with AKAP3, a major constituent of the fibrous sheath of the sperm tail. The successful large-scale production of recombinant mammalian OVGP1s can now pave the way for further identification and characterization of capacitation-associated and OVGP1-enhanced tyrosine phosphorylated proteins, and for further elucidation of their role in fertilization and development of early embryos. It is noteworthy to mention that, along with the use of molecular and biochemical techniques, the use of various immunohistochemical and immunocytochemical methods have contributed to our current understanding of the synthesis, secretion, and localization of OVGP1 in various mammalian species at both light and electron microscopic levels. Future studies are warranted to investigate the mechanisms that regulate the physiological functions of OVGP1. Since OVGP1 identified in various mammalian species is glycosylated, it will be of interest to examine the role of glycan derivatives of OVGP1 in early events of fertilization. It is anticipated that the use of various immunohistochemical and immunocytochemical investigative tools in conjunction with biochemical and molecular techniques will continue to be valuable investigative tools in advancing our knowledge of the importance of OVGP1 in reproductive functions.
